# Supplementary material for: A proposed methodology for uncertainty extraction and verification in priority setting partnerships with the James Lind Alliance: an example from the Common Conditions Affecting the Hand and Wrist Priority Setting Partnership
Source: BMC Med Res Methodol. 2022 Nov 10;22:292. doi: 10.1186/s12874-022-01777-5 (PMC9650903; doi:10.1186/s12874-022-01777-5)
Supplement: Supplementary file 2 — Additional file 2: Supplementary file 2. Included records, deemed within scope and submitted for research uncertainty extraction. [file 12874_2022_1777_MOESM2_ESM.pdf]

Supplementary file 2: Included records, deemed within scope and submitted for research uncertainty extraction

__Cochrane Reviews__

n = 45

Carpal tunnel syndrome

Surgical treatment options for carpal tunnel syndrome.
Cochrane Database of Systematic Reviews, no. 4 (2007)
http://onlinelibrary.wiley.com/doi/10.1002/14651858.CD003905.pub3/abstract

Surgical versus non-surgical treatment for carpal tunnel syndrome.
Cochrane Database of Systematic Reviews, no. 4 (2008)
http://onlinelibrary.wiley.com/doi/10.1002/14651858.CD001552.pub2/abstract

Endoscopic release for carpal tunnel syndrome.
Cochrane Database of Systematic Reviews, no. 1 (2014)
http://onlinelibrary.wiley.com/doi/10.1002/14651858.CD008265.pub2/abstract

Splinting for carpal tunnel syndrome.
Cochrane Database of Systematic Reviews, no. 7 (2012)
http://onlinelibrary.wiley.com/doi/10.1002/14651858.CD010003/abstract

Local corticosteroid injection for carpal tunnel syndrome.
Cochrane Database of Systematic Reviews, no. 2 (2007)
http://onlinelibrary.wiley.com/doi/10.1002/14651858.CD001554.pub2/abstract

Non-surgical treatment (other than steroid injection) for carpal tunnel syndrome.
Cochrane Database of Systematic Reviews, no. 1 (2003)
http://onlinelibrary.wiley.com/doi/10.1002/14651858.CD003219/abstract

Therapeutic ultrasound for carpal tunnel syndrome.
Cochrane Database of Systematic Reviews, no. 3 (2013)
http://onlinelibrary.wiley.com/doi/10.1002/14651858.CD009601.pub2/abstract

Rehabilitation following carpal tunnel release.
Cochrane Database of Systematic Reviews, no. 2 (2016)
http://onlinelibrary.wiley.com/doi/10.1002/14651858.CD004158.pub3/abstract

Exercise and mobilisation interventions for carpal tunnel syndrome.
Cochrane Database of Systematic Reviews, no. 6 (2012)
http://onlinelibrary.wiley.com/doi/10.1002/14651858.CD009899/abstract

Ergonomic positioning or equipment for treating carpal tunnel syndrome.

Cochrane Database of Systematic Reviews, no. 1 (2012)
http://onlinelibrary.wiley.com/doi/10.1002/14651858.CD009600/abstract

Cubital tunnel syndrome & ulnar neuropathy/nerve compression

Treatment for ulnar neuropathy at the elbow.
Cochrane Database of Systematic Reviews, no. 11 (2016)
http://onlinelibrary.wiley.com/doi/10.1002/14651858.CD006839.pub3/abstract

Compression neuropathies other than carpal/cubital tunnel

Treatment for thoracic outlet syndrome.
Cochrane Database of Systematic Reviews, no. 11 (2014)
http://onlinelibrary.wiley.com/doi/10.1002/14651858.CD007218.pub3/abstract

Stroke & adult spasticity

Hands-on therapy interventions for upper limb motor dysfunction following stroke.
Cochrane Database of Systematic Reviews, no. 6 (2011)
http://onlinelibrary.wiley.com/doi/10.1002/14651858.CD006609.pub2/abstract

Interventions for improving upper limb function after stroke.
Cochrane Database of Systematic Reviews, no. 11 (2014)
http://onlinelibrary.wiley.com/doi/10.1002/14651858.CD010820.pub2/abstract

Cerebral palsy

Surgical treatment for the thumb-in-palm deformity in patients with cerebral palsy.
Cochrane Database of Systematic Reviews, no. 4 (2005)
http://onlinelibrary.wiley.com/doi/10.1002/14651858.CD004093.pub2/abstract

De Quervain's disease

Corticosteroid injection for de Quervain's tenosynovitis.
Cochrane Database of Systematic Reviews, no. 3 (2009)
http://onlinelibrary.wiley.com/doi/10.1002/14651858.CD005616.pub2/abstract

Dupuytren's contracture

Surgery for Dupuytren's contracture of the fingers.
Cochrane Database of Systematic Reviews, no. 12 (2015)

http://onlinelibrary.wiley.com/doi/10.1002/14651858.CD010143.pub2/abstract

Trigger digits

Corticosteroid injection for trigger finger in adults.
Cochrane Database of Systematic Reviews, no. 1 (2009)
http://onlinelibrary.wiley.com/doi/10.1002/14651858.CD005617.pub2/abstract

Osteoarthritis of the hand

Surgery for thumb (trapeziometacarpal joint) osteoarthritis.
Cochrane Database of Systematic Reviews, no. 2 (2015)
http://onlinelibrary.wiley.com/doi/10.1002/14651858.CD004631.pub4/abstract

Inflammatory conditions and arthropathies

Rheumatoid arthritis & wrist arthroplasty

Occupational therapy for rheumatoid arthritis.
Cochrane Database of Systematic Reviews, no. 1 (2004)
http://onlinelibrary.wiley.com/doi/10.1002/14651858.CD003114.pub2/abstract

Splints and orthosis for treating rheumatoid arthritis.
Cochrane Database of Systematic Reviews, no. 4 (2001)
http://onlinelibrary.wiley.com/doi/10.1002/14651858.CD004018/abstract

Post-operative therapy for metacarpophalangeal arthroplasty.
Cochrane Database of Systematic Reviews, no. 1 (2008)
http://onlinelibrary.wiley.com/doi/10.1002/14651858.CD003522.pub2/abstract

Gout

Interventions for tophi in gout.
Cochrane Database of Systematic Reviews, no. 10 (2014)
http://onlinelibrary.wiley.com/doi/10.1002/14651858.CD010069.pub2/abstract

Distal radius fractures

Conservative interventions for treating distal radial fractures in adults.
Cochrane Database of Systematic Reviews, no. 2 (2003)
http://onlinelibrary.wiley.com/doi/10.1002/14651858.CD000314/abstract

External fixation versus conservative treatment for distal radial fractures in adults.
Cochrane Database of Systematic Reviews, no. 3 (2007)
http://onlinelibrary.wiley.com/doi/10.1002/14651858.CD006194.pub2/abstract

Different methods of external fixation for treating distal radial fractures in adults.
Cochrane Database of Systematic Reviews, no. 1 (2008)
http://onlinelibrary.wiley.com/doi/10.1002/14651858.CD006522.pub2/abstract

Percutaneous pinning for treating distal radial fractures in adults.
Cochrane Database of Systematic Reviews, no. 3 (2007)
http://onlinelibrary.wiley.com/doi/10.1002/14651858.CD006080.pub2/abstract

Pin site care for preventing infections associated with external bone fixators and pins.
Cochrane Database of Systematic Reviews, no. 12 (2013)
http://onlinelibrary.wiley.com/doi/10.1002/14651858.CD004551.pub3/abstract

Closed reduction methods for treating distal radial fractures in adults.
Cochrane Database of Systematic Reviews, no. 1 (2003)
http://onlinelibrary.wiley.com/doi/10.1002/14651858.CD003763/abstract

Bone grafts and bone substitutes for treating distal radial fractures in adults.
Cochrane Database of Systematic Reviews, no. 2 (2008)
http://onlinelibrary.wiley.com/doi/10.1002/14651858.CD006836.pub2/abstract

Rehabilitation for distal radial fractures in adults.
Cochrane Database of Systematic Reviews, no. 9 (2015)
http://onlinelibrary.wiley.com/doi/10.1002/14651858.CD003324.pub3/abstract

Anaesthesia for treating distal radial fracture in adults.
Cochrane Database of Systematic Reviews, no. 3 (2002)
http://onlinelibrary.wiley.com/doi/10.1002/14651858.CD003320/abstract

Carpal pathology

Computed tomography versus magnetic resonance imaging versus bone scintigraphy for clinically suspected scaphoid fractures in patients with negative plain radiographs.
Cochrane Database of Systematic Reviews, no. 6 (2015)
http://onlinelibrary.wiley.com/doi/10.1002/14651858.CD010023.pub2/abstract

Metacarpal fractures & injuries

Conservative treatment for closed fifth (small finger) metacarpal neck fractures.
Cochrane Database of Systematic Reviews, no. 3 (2005)
http://onlinelibrary.wiley.com/doi/10.1002/14651858.CD003210.pub3/abstract

Digital fractures & injuries

Conservative interventions for treating hyperextension injuries of the proximal interphalangeal joints of the fingers.
Cochrane Database of Systematic Reviews, no. 2 (2013)
http://onlinelibrary.wiley.com/doi/10.1002/14651858.CD009030.pub2/abstract

Interventions for treating mallet finger injuries.
Cochrane Database of Systematic Reviews, no. 3 (2004)
http://onlinelibrary.wiley.com/doi/10.1002/14651858.CD004574.pub2/abstract

Antibiotics for preventing infection in open limb fractures.
Cochrane Database of Systematic Reviews, no. 1 (2004)
http://onlinelibrary.wiley.com/doi/10.1002/14651858.CD003764.pub2/abstract
Full text has phalangeal fractures in the hand as a planned subgroup for the analysis, and three included studies were limited to open fractures of fingers.

Microvascular reconstruction

Low molecular weight heparin for prevention of microvascular occlusion in digital replantation.
Cochrane Database of Systematic Reviews, no. 7 (2013)
http://onlinelibrary.wiley.com/doi/10.1002/14651858.CD009894.pub2/abstract

Complex regional pain syndrome and neuropathic pain

Interventions for treating pain and disability in adults with complex regional pain syndrome- an overview of systematic reviews.
Cochrane Database of Systematic Reviews, no. 4 (2013)
http://onlinelibrary.wiley.com/doi/10.1002/14651858.CD009416.pub2/abstract

Local anaesthetic sympathetic blockade for complex regional pain syndrome.
Cochrane Database of Systematic Reviews, no. 7 (2016)
http://onlinelibrary.wiley.com/doi/10.1002/14651858.CD004598.pub4/abstract

Physiotherapy for pain and disability in adults with complex regional pain syndrome (CRPS) types I and II.
Cochrane Database of Systematic Reviews, no. 2 (2016)
http://onlinelibrary.wiley.com/doi/10.1002/14651858.CD010853.pub2/abstract

Other hand trauma

Vocational rehabilitation for enhancing return-to-work in workers with traumatic upper limb injuries.
Cochrane Database of Systematic Reviews, no. 10 (2013)

http://onlinelibrary.wiley.com/doi/10.1002/14651858.CD010002.pub2/abstract

Antibiotic prophylaxis for mammalian bites.
Cochrane Database of Systematic Reviews, no. 2 (2001)
http://onlinelibrary.wiley.com/doi/10.1002/14651858.CD001738/abstract
Full text indicates hand bites included, and included studies and conclusions include hand-specific content and differences from other bite sites.

Primary closure versus delayed closure for non bite traumatic wounds within 24 hours post injury
Cochrane Database of Systematic Reviews, no. 10 (2013)
http://onlinelibrary.wiley.com/doi/10.1002/14651858.CD008574.pub3/abstract

Anaesthesia and analgesia

Adrenaline with lidocaine for digital nerve blocks.
Cochrane Database of Systematic Reviews, no. 3 (2015)
http://onlinelibrary.wiley.com/doi/10.1002/14651858.CD010645.pub2/abstract

Protocols

n = 18

Infection & antibiotic use

Peri-operative antibiotics for hand trauma involving tendons and nerves.
Cochrane Database of Systematic Reviews, no. 6 (2012)
http://onlinelibrary.wiley.com/doi/10.1002/14651858.CD002107.pub2/abstract

Carpal tunnel syndrome

Acupuncture and related interventions for the treatment of symptoms associated with carpal tunnel syndrome.
Cochrane Database of Systematic Reviews, no. 7 (2014)
http://onlinelibrary.wiley.com/doi/10.1002/14651858.CD011215/abstract

Open release for carpal tunnel syndrome.
Cochrane Database of Systematic Reviews, no. 3 (2014)
http://onlinelibrary.wiley.com/doi/10.1002/14651858.CD011041/abstract

Absorbable versus non-absorbable sutures for carpal tunnel release.
Cochrane Database of Systematic Reviews, no. 6 (2015)
http://onlinelibrary.wiley.com/doi/10.1002/14651858.CD011757/abstract

Stroke & adult spasticity

Assistive technology, including orthotic devices, for the management of contractures in adult stroke patients.
Cochrane Database of Systematic Reviews, no. 10 (2013)
http://onlinelibrary.wiley.com/doi/10.1002/14651858.CD010779/abstract

Physical treatment interventions for managing spasticity after stroke.
Cochrane Database of Systematic Reviews, no. 7 (2011)
http://onlinelibrary.wiley.com/doi/10.1002/14651858.CD009188/abstract

Other nerve disorders

Dystonia

Botulinum a toxin for focal hand dystonia.
Cochrane Database of Systematic Reviews, no. 4 (2015)

http://onlinelibrary.wiley.com/doi/10.1002/14651858.CD004556.pub2/abstract

Dupuytren's contracture

Rehabilitation after surgery for Dupuytren's contracture.
Cochrane Database of Systematic Reviews, no. 2 (2007)
http://onlinelibrary.wiley.com/doi/10.1002/14651858.CD006508/abstract

Trigger digits

Surgery for trigger finger.
Cochrane Database of Systematic Reviews, no. 6 (2012)
http://onlinelibrary.wiley.com/doi/10.1002/14651858.CD009860/abstract

Osteoarthritis of the hand

Exercises for hand osteoarthritis.
Cochrane Database of Systematic Reviews, no. 2 (2013)
http://onlinelibrary.wiley.com/doi/10.1002/14651858.CD010388/abstract

Inflammatory conditions and arthropathies

Rheumatoid arthritis & wrist arthroplasty

Exercise therapy for the rheumatoid hand.
Cochrane Database of Systematic Reviews, no. 4 (2012)
http://onlinelibrary.wiley.com/doi/10.1002/14651858.CD003832.pub2/abstract

Space-occupying lesions

Ganglia

Interventions for ganglion cysts in adults.
Cochrane Database of Systematic Reviews, no. 2 (2005)
http://onlinelibrary.wiley.com/doi/10.1002/14651858.CD005327/abstract

Distal radius fractures

Internal fixation for treating distal radius fractures in adults.
Cochrane Database of Systematic Reviews, no. 7 (2014)
http://onlinelibrary.wiley.com/doi/10.1002/14651858.CD011213/abstract

Internal fixation versus other surgical methods for treating distal radius fractures in adults.
Cochrane Database of Systematic Reviews, no. 7 (2014)
http://onlinelibrary.wiley.com/doi/10.1002/14651858.CD011212/abstract

Carpal pathology

Scaphoid fractures

Surgical versus conservative interventions for treating acute scaphoid fractures in adults.
Cochrane Database of Systematic Reviews, no. 6 (2014)
http://onlinelibrary.wiley.com/doi/10.1002/14651858.CD010035.pub2/abstract

Conservative interventions for treating scaphoid fractures in adults.
Cochrane Database of Systematic Reviews, no. 8 (2013)
http://onlinelibrary.wiley.com/doi/10.1002/14651858.CD010713/abstract

Digital fractures & injuries

Interventions for treating ulnar collateral ligament injuries of the thumb.
Cochrane Database of Systematic Reviews, no. 8 (2014)
http://onlinelibrary.wiley.com/doi/10.1002/14651858.CD011267/abstract

Other hand trauma

Complex regional pain syndrome and neuropathic pain

Vitamin C for preventing complex regional pain syndrome (Type I) after wrist fractures in adults.
Cochrane Database of Systematic Reviews, no. 12 (2015)
http://onlinelibrary.wiley.com/doi/10.1002/14651858.CD007817.pub2/abstract

Guidelines

n= 16

Trigger digits

BSSH: Evidence based management of adult trigger digits

Stroke & adult spasticity

NICE: Stroke rehabilitation in adults : guidance (CG162)

SIGN: Guideline 118: Management of patients with stroke: rehabilitation, prevention and management of complications, and discharge planning - Full guideline [PDF]

Dupuytren's contracture

NICE: Radiation therapy for early Dupuytren's disease - guidance (IPG368)

NICE: Needle fasciotomy for Dupuytren's contracture - guidance (IPG43)

Osteoarthritis

NICE: Osteoarthritis: care and management : guidance (CG177)

NICE: Artificial metacarpophalangeal and interphalangeal joint replacement for end-stage arthritis - guidance (IPG110)

NICE: Artificial trapeziometacarpal joint replacement for end-stage osteoarthritis - guidance (IPG111)

Rheumatoid arthritis & wrist arthroplasty

NICE: Rheumatoid arthritis in adults: management : guidance (CG79)

NICE: Total wrist replacement - guidance (IPG271)

Hand fractures

NICE: Fractures (non-complex): assessment and management : guidance (NG38)

NICE: Fractures (complex): assessment and management : guidance (NG37)

NICE: Low-intensity pulsed ultrasound to promote fracture healing - guidance (IPG374)

Hand transplantation

NICE: Hand allotransplantation - guidance (IPG383)

Prostheses

NICE: Direct skeletal fixation of limb or digit prostheses using intraosseous transcutaneous implants - guidance (IPG270)

Brachial plexus injury

NICE: Phrenic nerve transfer in brachial plexus injury - guidance (IPG468)
